# Resolving Heterogeneity in Posttraumatic Stress Disorder Using Individualized Structural Covariance Network Analysis

**DOI:** 10.1155/2024/4399757

**Published:** 2024-11-01

**Authors:** Xueling Suo, Nanfang Pan, Li Chen, Lingjiang Li, Graham J. Kemp, Song Wang, Qiyong Gong

**Affiliations:** ^1^Department of Radiology, Huaxi MR Research Center (HMRRC), Institution of Radiology and Medical Imaging, West China Hospital of Sichuan University, Chengdu 610041, Sichuan, China; ^2^Functional and Molecular lmaging Key Laboratory of Sichuan Province, West China Hospital of Sichuan University, Chengdu 610041, Sichuan, China; ^3^Research Unit of Psychoradiology, Chinese Academy of Medical Sciences, Chengdu 610041, Sichuan, China; ^4^Mental Health Institute, The Second Xiangya Hospital of Central South University, Changsha 410008, China; ^5^Liverpool Magnetic Resonance Imaging Centre (LiMRIC) and Institute of Life Course and Medical Sciences, University of Liverpool, Liverpool L69 3GE, UK; ^6^Xiamen Key Lab of Psychoradiology and Neuromodulation, Department of Radiology, West China Xiamen Hospital of Sichuan University, Xiamen 361022, Fujian, China

**Keywords:** heterogeneity, individual differential structural covariance network, psychoradiology, PTSD, subtypes

## Abstract

The heterogeneity of posttraumatic stress disorder (PTSD) is an obstacle to both understanding and therapy, and this has prompted a search for internally homogeneous neuroradiological subgroups within the broad clinical diagnosis. We set out to do this using the individual differential structural covariance network (IDSCN). We constructed cortical thickness-based IDSCN using T1-weighted images of 89 individuals with PTSD (mean age 42.8 years, 60 female) and 89 demographically matched trauma-exposed non-PTSD (TENP) controls (mean age 43.1 years, 63 female). The IDSCN metric quantifies how the structural covariance edges in a patient differ from those in the controls. We examined the structural diversity of PTSD and variation among subtypes using a hierarchical clustering analysis. PTSD patients exhibited notable diversity in distinct structural covariance edges but mainly affecting three networks: default mode, ventral attention, and sensorimotor. These changes predicted individual PTSD symptom severity. We identified two neuroanatomical subtypes: the one with higher PTSD symptom severity showed lower structural covariance edges in the frontal cortex and between frontal, parietal, and occipital cortex—regions that are functionally implicated in selective attention, response selection, and learning tasks. Thus, deviations in structural covariance in large-scale networks are common in PTSD but fall into two subtypes. This work sheds light on the neurobiological mechanisms underlying the clinical heterogeneity and may aid in personalized diagnosis and therapeutic interventions.

## 1. Introduction

The clinical heterogeneity of posttraumatic stress disorder (PTSD) is a significant obstacle to both understanding and therapy, evident in the diversity of symptom presentations, genetic and epigenetic variation, and treatment response [1–3]. If this heterogeneity is driven by neurobiology, then identifying more internally homogeneous, biological subgroups within the broad clinical diagnosis may help disentangle it. Characterizing PTSD subtypes has typically involved identifying clusters of symptoms or epigenetics and testing these for distinct neurophysiological patterns [3, 4]. Recent evidence that symptoms and treatment response are both connected to impaired coordination among large-scale brain networks [5–7] suggests that neurobiological phenotyping based on disrupted brain networks may capture functionally relevant diversity [8], with a good chance of predicting treatment response and hence guiding treatment strategy [9].

What has become known as psychoradiology offers powerful tools to explore brain networks [10], which not only contribute to unraveling the intricacies of the brain but also hold promise in revolutionizing psychiatric research and clinical practice by offering quantitative and objective measures for assessing mental health conditions. Functional network-based analysis using resting-state functional magnetic resonance imaging (MRI) and electroencephalography yields PTSD neuroradiological subtypes with some ability to predict response to psychotherapy [11, 12]. However, structural covariance connectivity features are more stable over time while sensitive to maturation and clinical traits [13]. Brain structure networks capture the common variation in the morphological characteristics of different brain regions, which is genetically heritable, linked to behavioral and cognitive abilities, and influenced by normal development, aging, and psychiatric conditions [14].

Several studies have characterized brain structural covariance networks (SCNs) in PTSD [15–17]. Most have focused on the group-level SCN [15, 17], limiting the ability to detect subject-level differences. Studies that have constructed subject-level networks of structural covariance in PTSD [16] have typically relied on the conventional case–control designs to investigate group differences, disregarding individual variations; this is a general weakness of case–control designs in psychiatric biomarker discovery, given the high neurobiological heterogeneity in current clinical diagnoses [18]. Recently proposed as a way to quantify individual structural variation, individualized differential SCN (IDSCN) analysis has identified distinct neuroanatomical subtypes of heterogeneous diagnostic groups such as schizophrenia, depression, and obsessive–compulsive disorder [19–22]. The strength of the IDSCN over traditional SCNs is that it is constructed at the individual level, taking into account information from the reference group, and explicitly reflects the degree of morphological variability in brain regions of each patient relative to that of the reference [22]. IDSCN has been able to uncover individualized differences that would be otherwise obscured by group-level analysis [19, 20]. Thus, defining individualized structural differences may help us to discover neuroimaging substrates underlying symptoms and uncover more homogeneous subtypes in PTSD.

**Figure 1:**
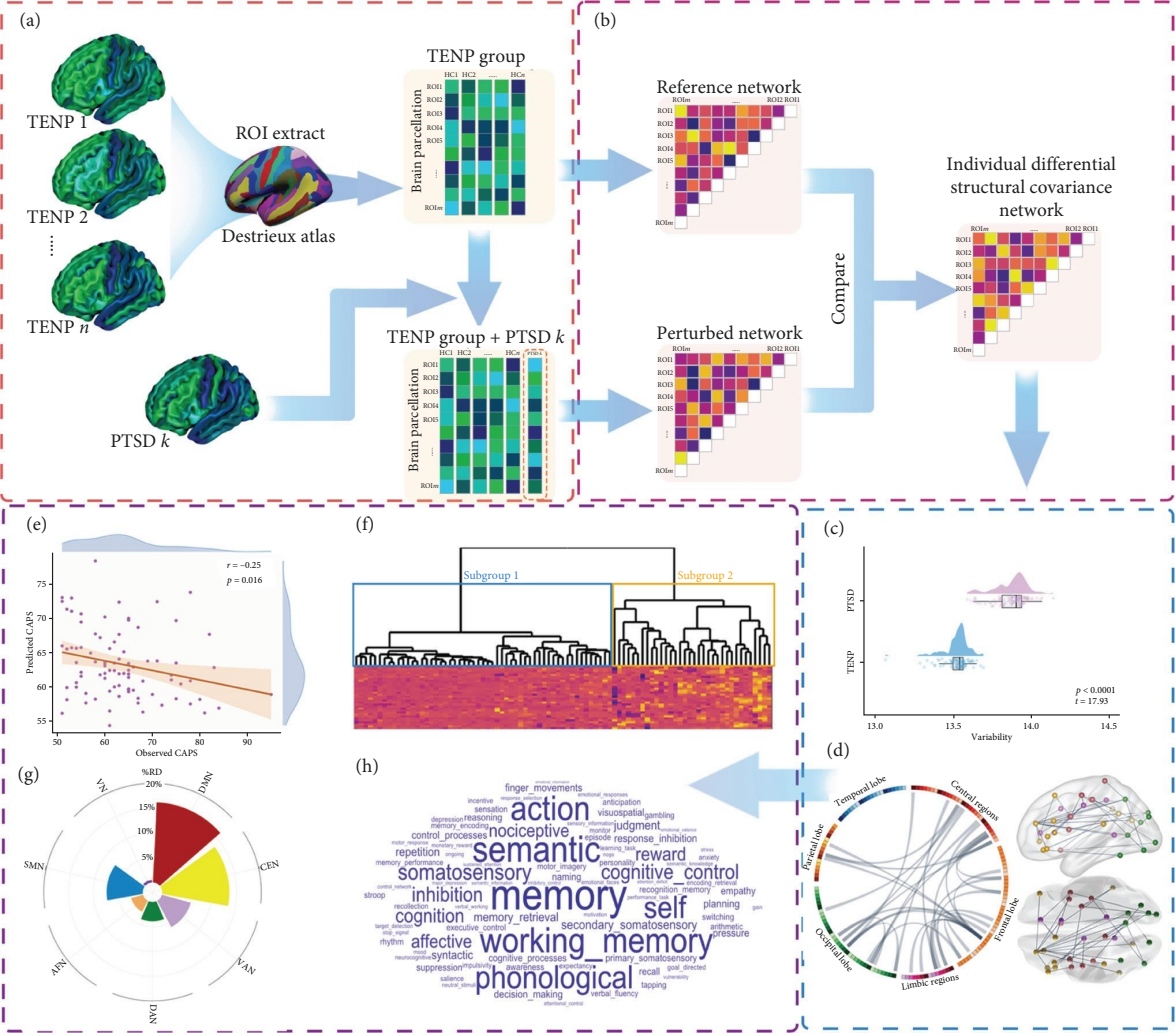
Flowchart for Individual Differential Structural Covariance Network (IDSCN) analysis. (a) Cortical thickness is calculated by surface-based morphometry and extracted based on Destrieux parcellation. (b) The reference network of structural covariance is constructed using Pearson correlation of cortical thickness between each pair of ROIs in the TENP control group, from which a perturbed network is created by adding PTSD patient *k*; the *Z*-score of the discrepancy between the perturbed and reference networks measures the IDSCN of patient *k*. (c) Heterogeneity comparison between the PTSD and TENP group uses the variability (Euclidean distance) between individualized SCN and group-level SCN. (d) The most differential edges are those different in at least 5% of the patients. (e) Predicted vs. observed CAPS scores show the performance of differential edges for symptom prediction using a random forest-based machine learning pipeline. (f) Subgroup clustering analysis uses a hierarchical tree; the height of each link in the dendrogram represents the distance between the clusters linked. (g) In the network distribution for differential edges shared by ≥5% of PTSD patients, the relative distribution (%RD) is the % of its overlapping voxels in a given network to the size of corresponding seed regions of differential edges. (h) In the functional annotation for differential edges, larger font size of functional terms corresponds to higher mean coactivation ratio (only terms surviving the permutation test [*p*  < 0.05] are shown). The scatterplots and word-clouds were created using R-based ggplot2 and wordcloud packages. For the circular ideograms of brain connectome, we utilized the circos toolbox in Perl, while the ball-and-stick charts were generated using the BrainNet Viewer toolbox in MATLAB. CAPS, Clinician-Administered PTSD Scale; PTSD, posttraumatic stress disorder; RD, relative distribution; ROI, region of interest; TENP, trauma-exposed non-PTSD.

We set out to use IDSCN analysis to explore structural covariance abnormalities in PTSD at the individual level, using as a reference trauma-exposed non-PTSD (TENP) controls to examine their correlations with PTSD symptom severity and to identify PTSD neuroanatomical subtypes (Figure 1). First, we investigated the extent of structural variability in PTSD, hypothesizing that, given the diverse symptoms and treatment responses, patients would exhibit higher heterogeneity than TENP controls. Then, we obtained individualized differential structural covariance edges for each patient, on the basis of which we clustered PTSD patients into subtypes and examined their neuroimaging and clinical characteristics. Finally, based on reports highlighting the potential sex effect on brain structure in PTSD [23, 24], we analyzed sex-by-subgroup interactions.

## 2. Materials and Methods

### 2.1. Participants

Survivors of a severe earthquake in Sichuan Province of China in 2008 were recruited between January and August 2009 and screened with the PTSD Checklist—Civilian Version (PCL-C), a 17-item self-reported measure of symptoms of PTSD [25]. In 8–15-month follow-up visits, the Structured Clinical Interview for the Diagnostic and Statistical Manual of Mental Disorders, fourth edition (DSM-IV) Diagnosis (SCID) [26] was used to confirm the diagnosis of PTSD, and the Clinician-Administered PTSD Scale (CAPS), a 30-item structured interview based on the DSM-IV, criteria for PTSD [27] to evaluate symptom severity by an experienced psychiatrist (L.L.). Survivors who scored PCL-C ≥ 35 and CAPS ≥ 50 were considered as PTSD if a diagnosis of PTSD was confirmed by SCID; those who scored PCL-C < 35 and did not meet the criteria for PTSD diagnosis by SCID were included in TENP controls [28, 29]. Inclusion criteria for all participants: (i) age ≥ 18 years; (ii) right-handed; (iii) physical experience of the earthquake; (iv) personal witness of building collapse, death, or serious injury; (v) no known PTSD prior to the earthquake; and (vi) no psychologic interventions or psychopharmacologic treatment before MRI. Exclusion criteria: age < 18 years; left-handedness; reported serious traumatic events before or after the earthquake; current and lifetime psychiatric comorbidities such as depression and anxiety disorders or alcohol/drug/other substance abuse/dependence; traumatic brain injury; neurological or cardiovascular conditions; any contraindication to MRI; brain lesions identified at MRI examination. The power analysis using G Power software [30] indicated that a sample of at least 128 participants would be needed to detect a medium-sized effect (Cohen's *d* = 0.5, *α* = 0.05, 1-*β* = 0.8) to conduct a two-sample *t*-test. Finally included were 89 PTSD patients who had not received treatment and had no psychiatric comorbidity and 89 demographically matched TENP controls. This recruitment strategy ensured that individuals with and without PTSD had similar earthquake experiences and demographic characteristics. Notably, several other analyses on these participants have been performed (e.g., analyses on cortical thickness [31], white matter microstructure [32], structural [33], and functional [34] network), with the results reported in the cited papers. The study protocol was reviewed and approved by the Sichuan University Research Ethics Committee. Each participant provided full-informed written consent. This study conforms to the provisions of the Declaration of Helsinki.

### 2.2. T1 Data Acquisition and Preprocessing

Participants underwent T1 structural imaging using a 3.0 T MR system (Excite; GE Healthcare, Milwaukee, WI) with an eight-channel phased-array head coil. Cushions and ear plugs stabilized the head. Acquisition used a spoiled gradient recalled sequence with an echo time of 3.4 ms, repetition time of 8.5 ms, flip angle 12°, slice thickness 1 mm, 156 axial slices, data matrix 256 × 256, and field of view 24 × 24 cm^2^. The total scanning time of each T1 structural run was about 5 min.

T1-weighted images were processed using FreeSurfer software (version 6.0) (http://surfer.nmr.mgh.harvard.edu/) with the standard recon-all stream. In brief, image processing comprised removing the skull, converting it to Montreal Neurological Institute (MNI) space, segmenting the brain into gray and white matter, normalizing intensity, creating a mesh of the boundary between white matter and gray matter, correcting topology, deforming and inflating surface, registering to a surface atlas, extracting the surface, and labeling gyri and sulci [35–37]. The outputs were inspected using the standard quality control procedures. Cortical thickness, determined as the shortest distance between the pial surface and the gray–white boundary [36], was extracted for 74 gray matter regions in each hemisphere from the Destrieux atlas [38] (chosen for homogeneity, reliability, and agreement with myeloarchitecture [39].

During scanning, each participant was asked to keep still and relaxed, close eyes, and not think of anything deliberately. Foam pads and earplugs were employed to reduce head motion and scanning noise. Before data preprocessing, each scanned file was inspected by an experienced neuroradiologist (X.S.) to rule out visible movement artifacts and gross structural abnormalities. During data preprocessing, two researchers (X.S. and N.P.) independently checked the quality of segmentation and normalization. According to these quality control procedures, no participants had to be excluded.

### 2.3. Constructing the IDSCN

IDSCN analysis was performed as follows [22]: To create the reference SCN (rSCN), the regional cortical thickness of each brain region was used to calculate Pearson correlations between all pairs of brain regions in all controls. For each patient (*k*, say), a perturbed SCN (pSCN) was built by including *k* with the controls to make a new group (Figure 1a). The difference between the perturbed and reference group SCN was calculated as *Δ*SCN = pSCN − rSCN, converted to a *Z*-score as follows:

  $$\begin{array}{c} Z=\frac{\Delta \text{SCN}}{\left(1-{\text{rSCN}}^{2}\right)/\left(n-1\right)}, \end{array}$$

where negative *Z* means lower structural covariance edge strength in *k* compared to controls, and vise versa. This *Z* was used to create the individual IDSCN for each patient *k* and converted to a *p*-value assuming the normal distribution. Lastly, for each patient, we identified individual variations in structural covariance edges that showed significant differences relative to rSCN with *p*  < 0.05, Bonferroni-corrected for 148 × 147/2 = 10,878 edges (Figure 1b). The IDSCN method has proven effective in studying individuals with depression, schizophrenia, and obsessive–compulsive disorder [19–22]. Its success lies in accurately pinpointing individual variations within psychiatric populations.

### 2.4. Heterogeneity of Individualized SCN and Group Difference

We investigated whether PTSD patients had higher morphological diversity than TENP controls by creating an *M* × *M* group-level SCN for each group, where *M* is the number of brain regions in the atlas. To obtain individualized SCN, a jackknife-bias estimation method was applied to assess the contribution of each subject to the overall group level SCN [40]. For each subject, the deviation between subject-level and group-level SCNs was measured as the Euclidean distance, and the morphological heterogeneity for a particular group was determined as its average. We compared heterogeneity between PTSD patients and TENP controls by two-sided two-sample *t* test (Figure 1c).

### 2.5. Identifying the Top Differential Edges

We first tallied the patients exhibiting significant alteration (*p*  < 0.05, Bonferroni-corrected) for each edge in the IDSCN. For further analysis, the top 32 covariance edges (Figure 1d) were chosen as features by virtue of their variation in ≥5% of patients (4/89 patients in this study) [22].

### 2.6. Linking IDSCN and Clinical Symptoms

We applied a machine learning pipeline based on the random forest algorithm (https://de.mathworks.com/matlabcentral/fileexchange/31036-random-forest) to predict the correlation between individualized differential structural covariance and clinical symptoms as measured by CAPS (Figure 1e) [41]. The mean *Z*-scores of the top 32 differential edges were used as input features. To ensure unbiased estimation of generalizability and prevent information leakage during model training, we implemented nested cross-validation with 10-fold splits in both inner and outer layers, repeating the procedure 10 times with random permutations of the data [42]. The optimal models selected from the inner layer were then applied to the test data in the outer layer, and their predictive performance was evaluated by mean-absolute error (MAE) and mean-squared error (MSE).

### 2.7. Subtyping PTSD Patients Using IDSCN

To assess the heterogeneity of structural covariance abnormalities among patients and to explore whether these tended to cluster in relatively homogeneous subgroups, we used hierarchical clustering to group subjects based on the similarity of their aberrant structural covariance patterns [43], using the top 32 edges as features. We used the NbClust package in R to determine the optimal number of clusters for our datasets (Figure 1f), and the agglomeration method was set to Ward's minimum variance with the Euclidean distance measure to associate individuals in the closest proximity and to evaluate dissimilarities between different entities [44]. The fundamental principle of hierarchical clustering algorithms entails iteratively merging or splitting clusters based on their proximity, ultimately constructing a hierarchical tree. This package provides 26 indices (e.g., Silhouette index, Calinski–Harabasz index, and Kullback–Liebler divergence) [44] to evaluate the quality of clustering results and indicate the optimum number of clusters (from 2 to 6) based on different criteria; here the most frequent value was selected as the optimal number of clusters. The Jaccard coefficient was used to assess the robustness of the cluster solution with bootstrapping (*n* = 1000), with clustering results with the Jaccard coefficient > 0.6 being considered stable [45].

### 2.8. Clinical and IDSCN Examination of PTSD Subtypes

We examined the differences between PTSD subtypes in terms of age, sex, education years, time since trauma, and total CAPS score with two-sided two-sample *t* test or chi-square test as appropriate. The two-sided two-sample *t* test was used to compare the top 32 differential edges between PTSD subtypes. Two-way analyses of variance were used to analyze PTSD subgroup × sex interactions; post hoc contrasts assessed the simple main effects when there were statistically significant interactions.

We mapped the case–control differential edges onto seven canonical brain networks based on the anatomical overlapping of seed regions [46]: the default mode network (DMN), central executive network (CEN), dorsal attention network (DAN), ventral attention network (VAN), cortical affective network (AFN), sensorimotor network (SMN), and visual network (VN). We then calculated the relative distribution (%RD) of each identified cluster within each network by assessing the ratio of its overlapping voxels in a given network to the size of corresponding seed regions of differential edges (Figure 1g) [47].

### 2.9. Functional Annotation of Altered Structural Covariance Edges

In order to understand the identified alterations of the structural covariance edges and to enhance their interpretability, we used functional annotation analysis (Figure 1h) incorporating Neurosynth datasets [21, 22] to explore their relationship with cognitive terms. Functional annotation analysis relies on probabilistic (activation) mapping, which provides quantitative inferences regarding the relationship between terms of cognitive processes and brain activity (Neurosynth, https://neurosynth.org/). The psychophysiological function of each region was interpreted by associating each voxel in an activation map with a certain number of terms/tasks [48]. From the Neurosynth datasets, we selected 217 terms with obvious biological importance [49]. We linked the distribution and annotation of the altered edges in each subtype to the functional/cognitive terms that survived permutation testing at *p*  < 0.05 [50], using Brain Annotation Toolbox (BAT, http://123.56.224.61/softwares) [50].

## 3. Results

### 3.1. Clinical Demographics

Table 1 shows the clinical and demographic characteristics of the participants. Age, sex, education years, and time since trauma showed no significant difference between PTSD and TENP (all *p*  > 0.05). Patients with PTSD had significantly higher PCL-C (mean 47.5 vs. 28.0, *p*  < 0.001) and CAPS (mean 63.6 vs. 22.5, *p*  < 0.001) scores than TENP.

### 3.2. Higher Heterogeneity in Patients With PTSD Than That in TENP

The variability between subject-level and group-level SCN was significantly higher in PTSD patients than in TENP controls, indicated by Euclidean distance (*p*  < 0.001, two-sided *t* = 17.93, Cohen's *d* = 2.69) (Figure S1).

### 3.3. Heterogeneity of IDSCN in PTSD

For each PTSD patient, we obtained distinct structural covariance edges from the reference network (*p*  < 0.05, Bonferroni-corrected for 10,878 edges). Patients with PTSD exhibited a wide and variable range of distinct edges. Of all the edges, 3492 (32%) were present in at least one patient and 831 (8%) in at least two patients. Figure S2 shows the number of differential edges in IDSCN for each subject and the number shared by subjects.

**Figure 2:**
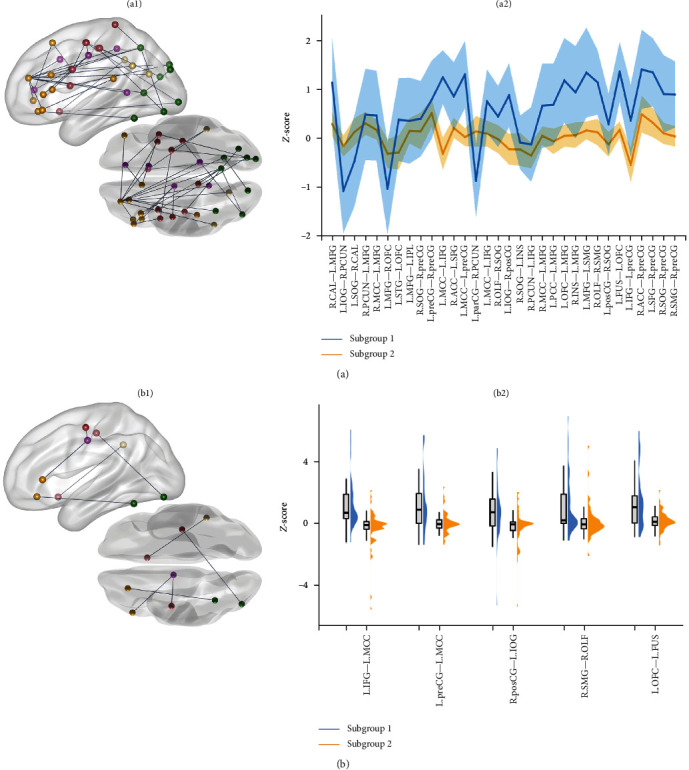
PTSD patients cluster into two subgroups. (a) The top 32 differential structural covariance edges (i.e., differential in at least 5% of patients) (a1) and their *Z*-score distribution in the 2 subgroups (a2). (b) The five edges that show significant difference (FDR corrected) between the two PTSD subgroups (b1) and their *Z*-score distribution in the two subgroups (b2). ACC, anterior cingulate cortex; CAL, calcarine sulcus; FUS, fusiform gyrus; IFG, inferior frontal gyrus; INS, insula; IOG, inferior occipital gyrus; IPL, inferior parietal gyrus; L, left; MCC, middle cingulate cortex; MFG, middle frontal gyrus; OFC, orbitofrontal cortex; OLF, olfactory cortex; parCG, aracentral gyrus; PCC, posterior cingulate cortex; PCUN, precuneus; posCG, postcentral gyrus; preCG, recentral gyrus; R, right; SFG, superior frontal gyrus; SMG, supramarginal gyrus; SOG, superior occipital gyrus; STG, superior temporal gyrus.

The 10,878 edges in the IDSCN were arranged in descending order based on the number of patients in which the edge is significantly altered. Patients were classified into two subgroups using the *Z*-score of the top 32 edges differing in at least four patients (i.e., ~5% of patients) as the classifying features (Figure 2a).

### 3.4. Association With Clinical Symptoms

To establish the relationship between clinical symptoms and the differential edges, we applied a random forest model to predict the total CAPS score for each patient using the mean *Z*-scores of the top 32 differential edges as input features. This could predict the total score of CAPS for all patients in the modeling (*r* = −0.25, *p* = 0.016, MAE = 8.7, MSE = 126.8) (Figure S3).

### 3.5. Subtypes of Patients With PTSD Identified by IDSCN

**Figure 3:**
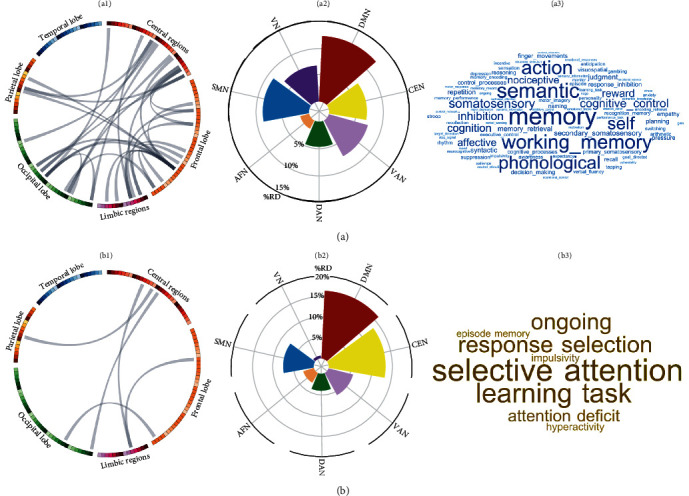
Brain connectome patterns, their network distribution and function annotation. (a) The top 32 differential structural covariance edges (a1) were mapped onto 7 canonical brain networks based on functional coactivation; the relative distribution (%RD) is the % of its overlapping voxels in a given network to the size of corresponding seed regions of differential edges (a2); in the functional associations (a3), larger font in the word-cloud corresponds to higher mean coactivation ratio (only terms surviving the permutation test [*p*  < 0.05] are shown). (b) Shows the same for the five edges which are significantly different between the two PTSD subgroups. The 5 edges (b1) were also mapped onto 7 canonical brain networks with their network relative distribution (b2) and functional associations (b3). AFN, cortical affective network; CEN, central executive network; DAN, dorsal attention network; DMN, default mode network; RD, relative distribution; SMN, sensorimotor network; VAN, ventral attention network; VN, visual network.

To subtype patients with PTSD, the top 32 edges shared by at least four patients (~5% of all patients) were selected as features. A two-cluster solution best captured the distinct and homogeneous subgroups of our datasets based on 26 criteria (Figure S4), and sample sizes of each cluster (subtype 1, *n* = 34; subtype 2, *n* = 55) were adequate to reveal significant differences between biotypes. The Jaccard coefficients were 0.754 for subtype 1, 0.850 for subtype 2. Of the top 32 edges, 5 showed significant differences between the 2 subtypes; these connected the left middle cingulate cortex with left inferior frontal gyrus and left precentral gyrus, the left orbital frontal gyrus with left fusiform, the right postcentral gyrus with left inferior occipital gyrus, as well as the right olfactory with right supramarginal gyrus, which showed higher *Z*-scores in subtype 1 relative to subtype 2 (Figure 2b). The two patient subtypes also showed significant differences in their symptom severity on CAPS (subtype 1, 61.1 ± 7.5; subtype 2, 65.2 ± 9.5, *p* = 0.038), but no significant differences in age, sex, years of education, time since trauma, or PCL-C score (all *p*  > 0.05). There was no significant PTSD subgroup × sex interaction in the top 32 edges (all *p*  > 0.05). The top 32 edges were primarily distributed in DMN, SMN, and VAN (Figure 3a and Table S1), while the top five edges were mainly in DMN, SMN, and CEN (Figure 3b and Table S1).

### 3.6. Functional Implications of the Covariance Edges

Out of the 217 terms, the top 32 edges in all patients were closely associated with functional terms (*p*  < 0.05 for permutation test) such as memory, semantic, and working memory (Figure 3a and Table S2), while subtype 1 exhibited distinct edges significantly linked to functional terms (*p*  < 0.05, permutation test) such as selective attention, response selection and learning task (Figure 3b and Table S3).

## 4. Discussion

As might be expected, the PTSD group had higher heterogeneity of structural covariance than TENP. Studying individualized structural covariance aberrance yielded three main results: (1) PTSD patients showed low overlap of altered edges, indicating high diversity in structural covariance. (2) Despite the notable brain-wide heterogeneity, the structural covariance edges constituting the DMN, SMN, and VAN were most often impacted, suggesting a common underlying influence. (3) Two neuroanatomical subtypes of PTSD were identified based on the shared structural covariance edges: patients who had more severe PTSD symptoms (type 2) showed less covariance between the frontal cortex (left middle cingulate cortex with left inferior frontal gyrus and left precentral gyrus), frontal and parietal/temporal cortex (right olfactory and right supramarginal gyrus, and left orbital frontal gyrus and left fusiform), as well as parietal and occipital cortex (right postcentral gyrus and left inferior occipital gyrus), which are mainly implicated in selective attention, response selection and learning task-related functional terms. These findings provide a new insight into a taxonomy that may help improve precision diagnosis and treatment for PTSD.

In previous approaches to this heterogeneity, latent class/profile analyses have been used to identify PTSD symptom typologies [51, 52] to help explain differences in treatment response [53]. However, symptom typologies often have overlapping neuroimaging patterns [54, 55] and are limited by imprecise diagnostic thresholds, which can either be insufficiently sensitive or fail to exclude subthreshold symptoms [56]. Neuroimaging data-driven approaches offer the hope of detecting more stable and homogeneous patient subtypes [12, 57], and indeed, PTSD subtypes have been identified using functional MRI [11] and electroencephalography (EEG) [12]. Here, we used this data-driven approach with structural covariance, which is likely to yield more stable trait-like connection features than functional approaches [13]. What is the biological significance of the brain morphological network? Morphological covariation or similarity may reflect coordinated development or synchronized maturation between brain regions [58]. Comparing individual brain morphological networks with classical cytoarchitectonic atlases, morphologically similar regions share similar cytoarchitectural biology [59]. The individual morphological similarity between cortical areas aligns with spatial expression patterns of certain important genes [59]. Although not all the details are understood, it is clear that genetic, cytoarchitectonic, and chemoarchitectonic factors play fundamental roles in forming and shaping the brain morphological network [60, 61]. We found that structural interindividual heterogeneity was very high in patients. This helps explain the inconsistent findings in earlier case–control structural covariance studies in PTSD. For instance, the frontal cortex, which shows high interindividual structural variability in the current study, was reported to have both increased and decreased structural connectivity in previous case–control studies [15, 17]. Our findings indicate that individualized structural covariance alteration is a key neuropathological characteristic of PTSD, and the alteration patterns among patients can be remarkably different.

Despite considerable variability in individual patient abnormalities, most patients showed common structural covariance edges involving DMN, SMN, and VAN. This broadly aligns with evidence that PTSD is associated with disruption to large-scale brain networks [5]. The DMN, comprising the medial prefrontal and posterior parietal cortices, is active in autobiographical memory, self-referential processing, and internal cognition [62], and disruptions in DMN are linked to deficits in emotion regulation and cognitive processes in PTSD patients, evident in persistent and exaggerated negative affective states, impaired emotional regulation and intrusion and dissociation symptoms [5]; all this supports DMN as a potential classification biomarker in PTSD [63]. The VAN, including the temporoparietal junction and ventral lateral prefrontal cortex, sometimes called the salience network [64], has been linked to internally directed cognition, which is important for mediating attentional subprocess [65, 66]. Earlier studies found that VAN may be predictive of treatment efficacy in PTSD, supporting a causal role and suggesting a potential targeting strategy [67]. While the large-scale, spatially distributed triple network model of PTSD is well established [7], based largely on functional neuroimaging findings, our structural covariance evidence about the SMN extends this by implicating sensorimotor involvement in the pathophysiological processes. Taken together, all these altered structural covariance edges enabled the prediction of individual PTSD symptom severity with encouraging accuracy.

We identified two neuroanatomical subtypes. The subgroup with more severe PTSD symptoms showed lower covariance within the frontal cortex (left middle cingulate cortex with left inferior frontal gyrus and left precentral gyrus), between the frontal and parietal/occipital regions (right olfactory and right supramarginal gyrus, and left orbital frontal gyrus and left fusiform), and between parietal and occipital cortex (right postcentral gyrus and left inferior occipital gyrus). The cingulate and inferior frontal gyrus play a crucial role in a broader network related to the selection and maintenance of mental representations [68]. The precentral and postcentral gyrus are important components of the SMN, which contributes to skill learning [69]; the lower structural covariance between these regions suggests that the subgroup with more severe PTSD symptoms may have more difficulty engaging the frontal network, which plays a crucial role in regulating thought content in working memory [68] and the SMN that is involved in preparation for coping with a physical threat [70]. Disruptions between these regions are more marked in PTSD subgroups with suicidal ideation [68], which respond to treatment [71], and with the stress-related gene FKBP5 diplotype [72]. The olfactory cortex is not commonly reported as affected in PTSD but may be related to olfactory deficits in PTSD [73, 74]. The orbitofrontal cortex is important in emotion modulation [75]; the visual cortex (fusiform and inferior occipital gyrus) has been implicated in identifying faces and perceiving facial expressions [76]. Structural covariance aberrance in the orbitofrontal/postcentral and visual cortex might be driven by specific symptom clusters, for example, aggressive/impulsive behaviors [77] and active avoidance in PTSD [78]. In addition, functional annotation analysis showed that dysfunction of these regions was closely related to selective attention, response selection, and learning tasks, in line with previous functional neuroimaging studies [79–81]. Most demographic and clinical characteristics showed no significant differences between these two subtypes, suggesting that they would be concealed by conventional group-level designs or symptom-based categories.

Heterogeneity hampers the identification of reliable neuroimaging markers indicative of precision in diagnosis and treatment [11]. Previously, sources of heterogeneity in PTSD neuroimaging studies have been attributed to multifactorial factors such as type of trauma, comorbid psychiatric disorders, medication status, and imaging protocols [82]. The current study showed that although these factors were well-controlled, PTSD patients still exhibited tremendous inter-individual heterogeneity. Moreover, we found subtypes with higher PTSD symptom severity showed lower structural covariance edges also involved in the prefrontal cortex and frontoparietal regions, which are functionally implicated in cognitive impairments. Individuals diagnosed with PTSD present substantial cognitive deficits in multiple domains, namely sustained attention, working memory, and memory [83]. In a pharmacological functional MRI study, tolcapone, a drug that enhances cortical dopamine tone via inhibition of the catechol-O-methyl transferase enzyme, improved working memory performance in individuals with more severe PTSD symptoms by upregulating the recruitment of frontoparietal regions [84]. Considering the remarkable difference in structural covariance aberrance, these two PTSD subtypes might have different treatment outcomes, which remains to be clarified in longitudinal studies. Altogether, our results highlight the prominent role of subtype research in understanding the neuroanatomical mechanism of heterogeneity in PTSD and reveal the potential in improving clinically precise diagnosis and optimizing treatment in the future.

The study has some limitations. First, this was one dataset and a single modality; future studies in independent datasets could use multimodal imaging methods incorporating neurofunctional metrics [85]. Second, it was a cross-sectional design; longitudinal research would be needed to establish whether these two PTSD subtypes showed different responses to treatment. Third, Neurosynth was used to examine the link between distinct structural covariance edges and cognitive terms. Individuals with PTSD also have cognitive impairments [86], which were not evaluated in the current study. Additionally, as we did not document the dimensional CAPS, the relation of symptom dimension with the identified subtypes could not be determined. Fourth, the purpose of our research was to determine structural covariance characteristics that distinguish stressed individuals who do and do not develop PTSD. Without a nontraumatized control group, we were unable to explore the heterogeneity of stress responses to trauma exposure. Fifth, a recent study identified epigenetic biotypes of PTSD based on DNA methylation profiles [3], and gene expression has been shown to be related to PTSD symptom clusters [87]. Future research with genetic data would offer more comprehensive insight into the biology of PTSD biotypes. Sixth, our sample was homogeneous in that all participants were exposed to the earthquake; however, one should be cautious in generalizing our findings to PTSD caused by other types of trauma. There was a possible effect of degree of exposure to the earthquake, for example physical location at the time of the attacks on brain responses [88]. A full study of different degrees of exposure to earthquakes will need a stratified analysis, which is beyond the scope of the current study, although it is a focus of ongoing work. Finally, due to the exploratory nature, the likelihood of our study being directly useful in clinical settings is limited.

## 5. Conclusion

Using an IDSCN and clustering analyses, this study identified two distinct neuroanatomical subtypes in PTSD. Although PTSD patients demonstrated remarkable inter-individual variability in individual structural covariance aberrance, common variability distributions were observed in DMN, VAN, and SMN, which supported and meanwhile extended the triple network mode of PTSD. While sharing most similar clinical and demographic characteristics, these two subtypes displayed differences in the structural covariance edges. In particular, the patient subgroup with more severe PTSD symptoms showed less covariance within the frontal cortex and between the frontal, parietal, and occipital cortex, which were mainly implicated in selective attention, response selection, and learning task-related functional terms. The two neuroanatomical subtypes we identified offer new insights into taxonomy, facilitate potential clues to precision diagnosis, and brain-based intervention of PTSD, such as transcranial magnetic stimulation, and more broadly, this work contributes to the development of psychoradiology [10].

## Figures and Tables

**Table 1:** Demographic and clinical characteristics of participants^a^.

Variables	TENP (*n* = 89)	PTSD (*n* = 89)	*p*
Age (years)^b^	43.1 ± 10.1 (20–65)	42.8 ± 10.7 (19–67)	0.85^c^
Sex (male/female)	26/63	29/60	0.75^d^
Years of education (years)^b^	6.9 ± 3.3 (0–12)	7.1 ± 3.1 (0–16)	0.74^c^
Time since trauma (months)^b^	11.7 ± 2.5 (8–15)	11.1 ± 2.3 (8–15)	0.11^c^
PCL-C score	28.0 ± 6.9 (18–54)	47.5 ± 12.5 (21–80)	<0.001^c^
CAPS score	22.5 ± 11.0 (3–48)	63.6 ± 9.0 (51–95)	<0.001^c^

Abbreviations: CAPS, Clinician-Administered PTSD Scale; PCL-C, PTSD Checklist—Civilian Version; PTSD, posttraumatic stress disorder; TENP, trauma-exposed non-PTSD control.

^a^Data are presented as mean ± SD (minimum–maximum) unless noted.

^b^Age, years of education, and time since trauma defined relative to the time of MRI.

^c^ *p* Using two-sided unpaired *t* test.

^d^ *p* Using two-sided Chi-squared test.

## Data Availability

The neuroimaging data in the present study are under active use by the reporting laboratory and are available from the corresponding author upon reasonable request. The probabilistic (activation) mappings are provided by Neurosynth (https://neurosynth.org/). All analytical procedures in this study are based on publicly available toolkits. Cortical thickness is assessed using FreeSurfer software (http://surfer.nmr.mgh.harvard.edu/). The script for the construction of IDSCN is in-house and available from the corresponding author upon request. Prediction analysis is performed using the built-in function in MATLAB 2018a and NeuroMiner toolbox (https://github.com/neurominer-git). Clustering analysis is performing based on NbClust (https://www.rdocumentation.org/packages/NbClust) and flexclust (https://cran.r-project.org/web/packages/flexclust) in R script. Functional annotation is performed using Brain Annotation Toolbox (BAT, http://123.56.224.61/softwares). Additional information is available from the corresponding author upon request.

## Supporting Information

Supporting Information Figure S1. Heterogeneity comparison between the PTSD and TENP group uses the variability (Euclidean distance) between individualized SCN and group-level SCN. Figure S2. Heterogeneity of IDSCN in patients with PTSD. Figure S3. Prediction performance of differential edges for CAPS scores. Figure S4. Clustering analysis. Table S1. %RD of each identified cluster within each network by assessing the % of its overlapping voxels in a given network to the size of corresponding seed regions of differential edges. Table S2. Functional annotation for the shared edges. Table S3. Functional annotation for the distinct edges. References for Supporting Information.
