# Supplementary material for: Resolving Heterogeneity in Posttraumatic Stress Disorder Using Individualized Structural Covariance Network Analysis
Source: Depress Anxiety. 2024 Nov 1;2024:4399757. doi: 10.1155/2024/4399757 (PMC11919208; doi:10.1155/2024/4399757)
Supplement: Supporting Information — Figure S1. Heterogeneity comparison between the PTSD and TENP group uses the variability (Euclidean distance) between individualized SCN and group-level SCN. Figure S2. Heterogeneity of IDSCN in patients with PTSD. Figure S3. Prediction performance of differential edges for CAPS scores. Figure S4. Clustering analysis. Table S1. %RD of each identified cluster within each network by assessing the % of its overlapping voxels in a given network to the size of corresponding seed regions of differential edges. Table S2. Functional annotation for the shared edges. Table S3. Functional annotation for the distinct edges. References for Supporting Information. [file 4399757.f1.docx]

**Supplementary Material for**

**‘Resolving heterogeneity in post-traumatic stress disorder using individualized structural covariance network’**

**Figure S1**. Heterogeneity comparison between the PTSD and TENP groups 2

**Figure S2**. Heterogeneity of IDSCN in patients with PTSD3

**Figure S3**. Prediction performance of differential edges for CAPS scores 4

**Figure S4**. Clustering analysis 5

**Table S1.** %RD of each identified cluster within each network 6

**Table S2.** Functional annotation for the shared edges 7

**Table S3**. Functional annotation for the distinct edges9

**References for Supplementary Materials**10


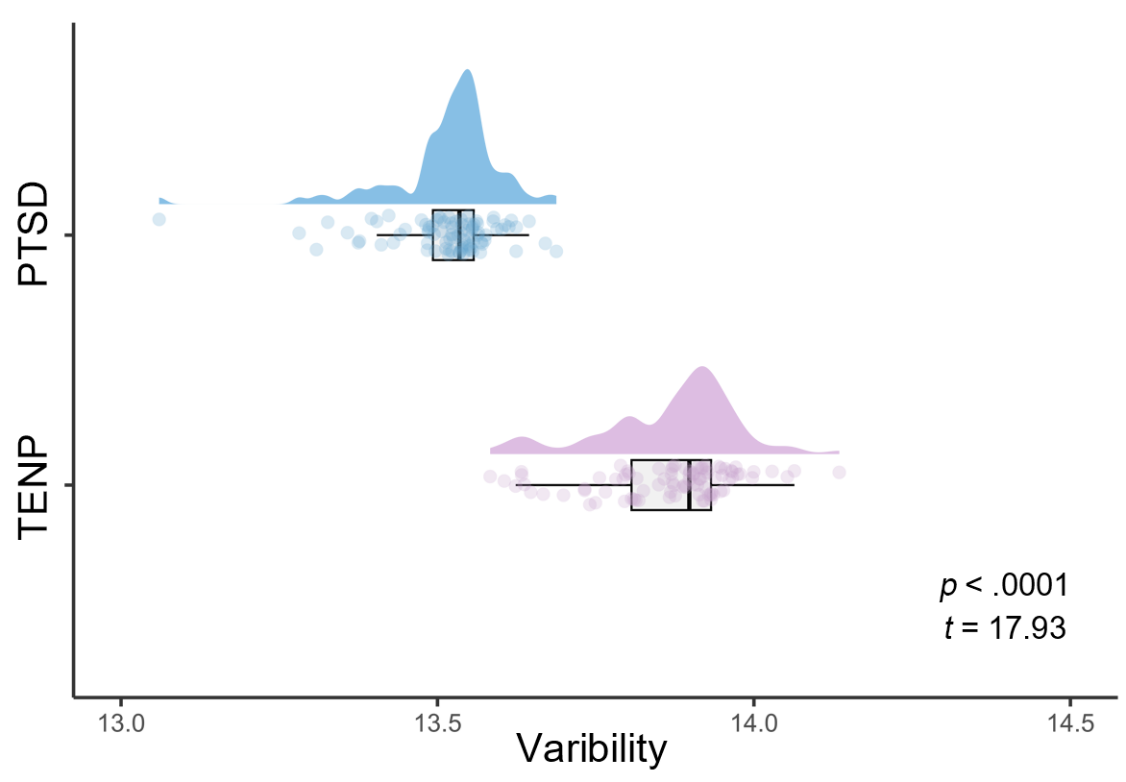


**Figure S1. Heterogeneity comparison between the PTSD and TENP groups.** This uses the variability (Euclidean distance) between individualized SCN and group-level SCN. Abbreviations: PTSD, posttraumatic stress disorder; TENP, trauma-exposed non PTSD.


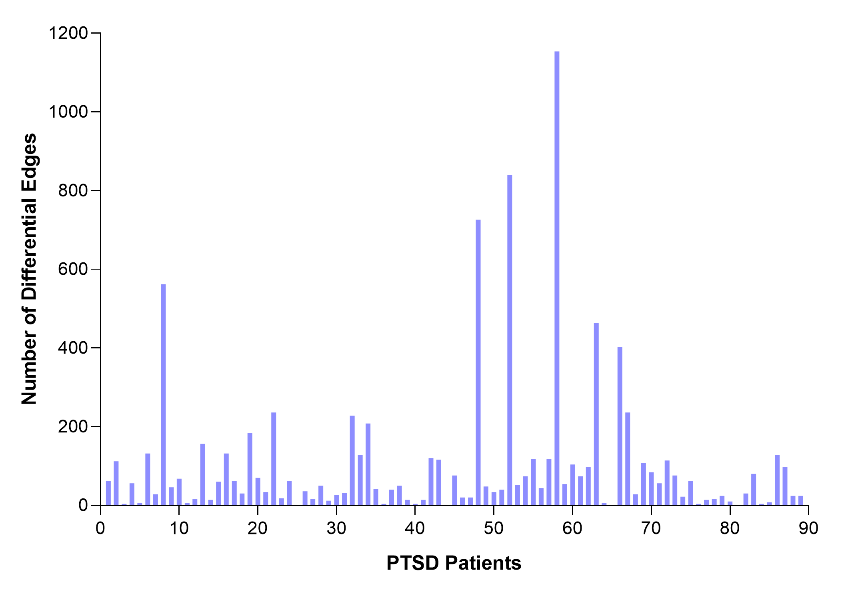


a


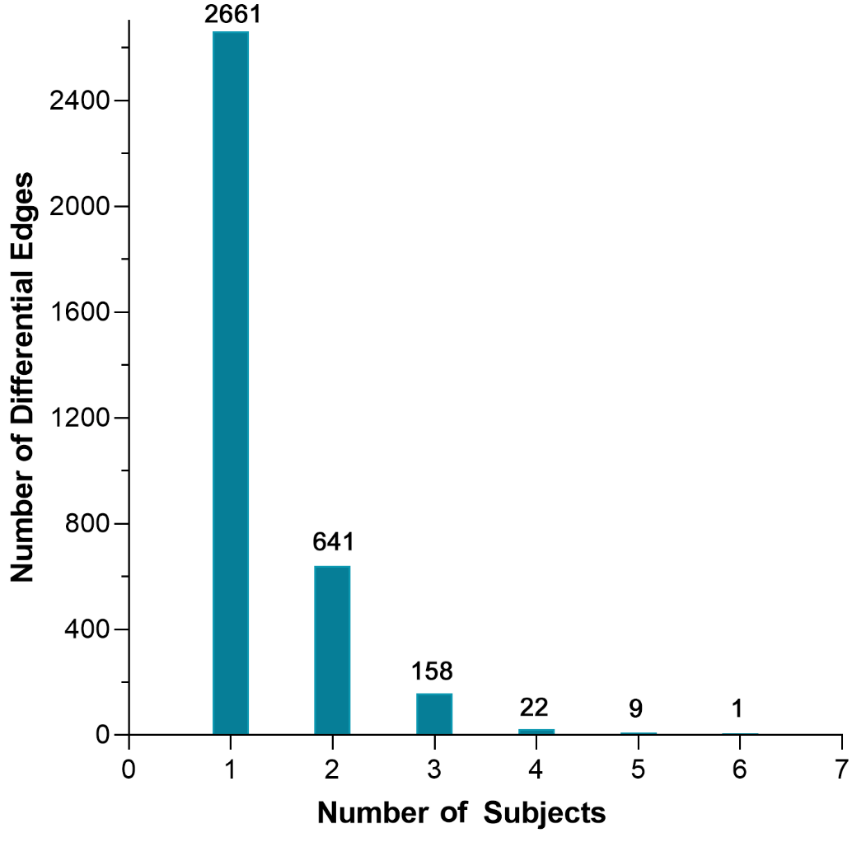


b

**Figure S2.** **Heterogeneity of IDSCN in patients with PTSD.** (a) Number of differential edges in IDSCN for each subject. (b) Number of differential edges in IDSCN shared by subjects. Among 10878 edges in total, 3492 edges are different in at least one subject, and the other 7386 edges are not deviated in any subjects. We chose these edges that were different in at least 4 patients (5% out of 89) as input features for clustering and machine learning pipeline. Abbreviations: PTSD, posttraumatic stress disorder; IDSCN, individual differential structural covariance network.


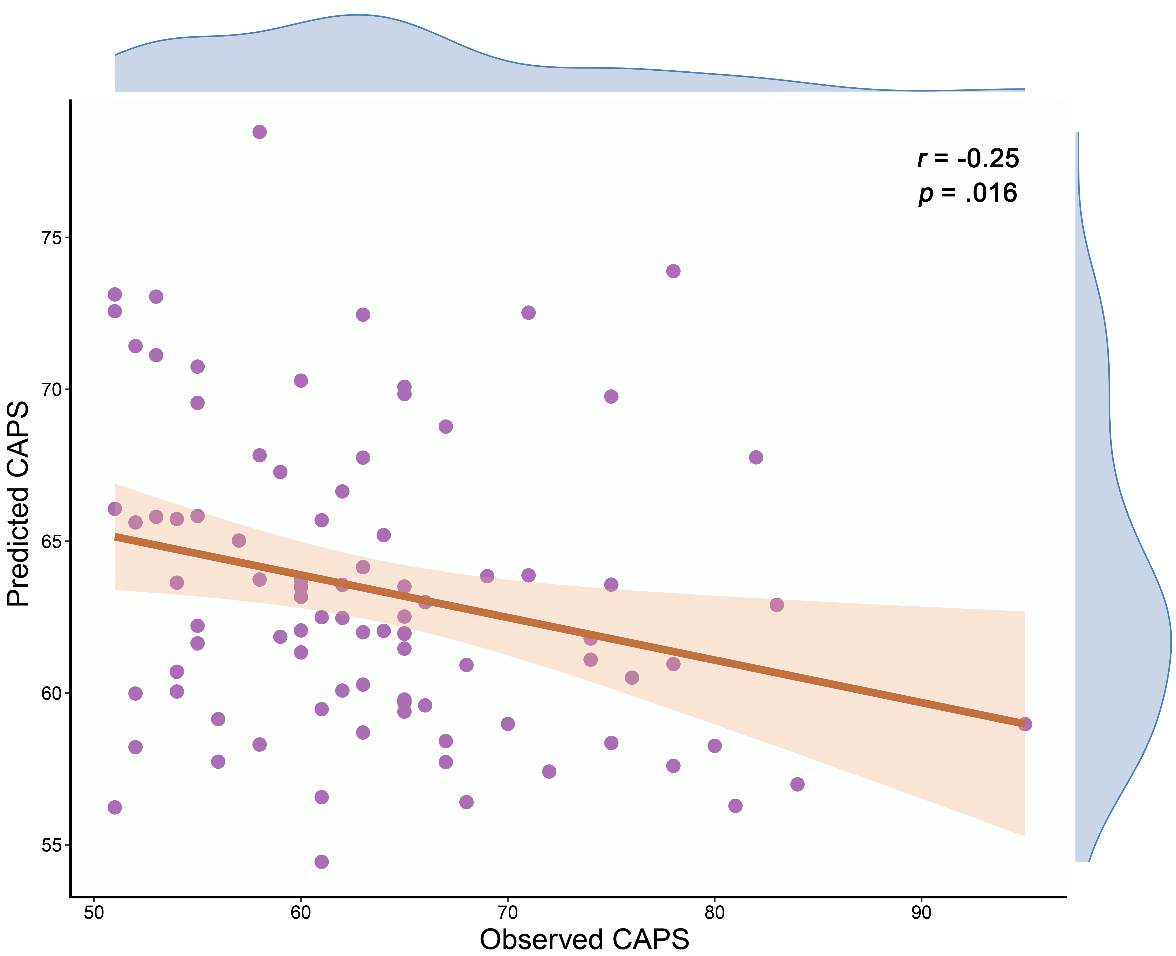


**Figure S3**. **Prediction performance of differential edges for CAPS scores.** Abbreviations: CAPS, Clinician-Administered PTSD Scale.


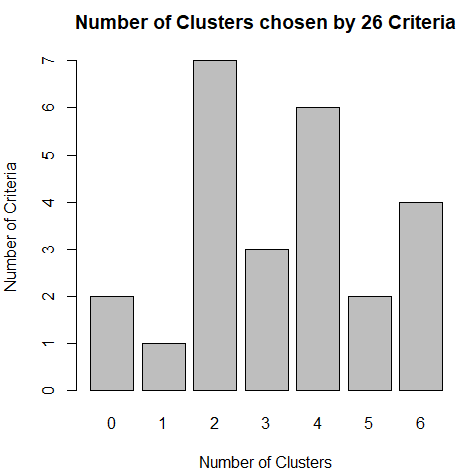


a


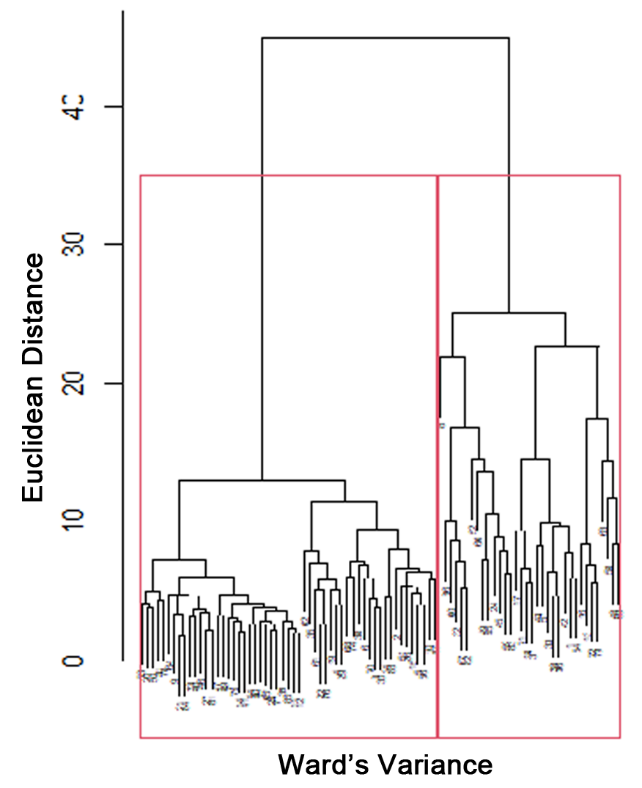


b

**Figure S4. Clustering analysis.** (a) Number of clusters chosen by 26 criteria. The following indices were calculated to determine the optimized number of clusters: kl, ch, hartigan, ccc, scott, marriot, trcovw, tracew, friedman, rubin, cindex, db, silhouette, duda, pseudot2, beale, ratkowsky, ball, ptbiserial, frey, mcclain, dunn, hubert, sdindex, dindex, and sdbw. See [1] for more details. (b) Hierarchical tree for clustering analysis. The height of each link in the resulting dendrogram represents the distance between the clusters being linked.

**Table S1.** %RD of each identified cluster.

| Overlap | %RD  case-control top 32 edges | %RD  5 edges that significantly differ across subgroups |
| --- | --- | --- |
| DMN | 12.18 | 16.20 |
| CEN | 6.96 | 13.52 |
| VAN | 7.51 | 6.02 |
| DAN | 4.64 | 3.90 |
| AFN | 1.56 | 2.48 |
| SMN | 8.55 | 7.29 |
| VN | 6.61 | 0.49 |

Note: %RD expresses the % of a cluster’s overlapping voxels in a given network relative to the size of corresponding seed regions of differential edges.

Abbreviations: DMN, default mode network; DAN, dorsal attention network; CEN, central executive network; AFN, cortical affective network; RD, relative distribution; SMN, sensorimotor network; VAN, ventral attention network; VN, visual network.

**Table S2. Functional annotation for the shared edges**

| Functional terms of Neurosynth | Mean Co-activation Ratio | Permutation P-value |
| --- | --- | --- |
| memory | 0.130462 | 0.008 |
| semantic | 0.092627 | 0.018 |
| working_memory * | 0.084981 | <0.001 |
| action | 0.081068 | 0.018 |
| phonological | 0.072075 | 0.007 |
| self | 0.066925 | 0.012 |
| somatosensory | 0.06494 | 0.043 |
| cognitive_control * | 0.057829 | <0.001 |
| inhibition * | 0.055617 | <0.001 |
| cognition | 0.050933 | 0.012 |
| reward | 0.047337 | 0.049 |
| affective | 0.045717 | 0.023 |
| nociceptive | 0.044226 | 0.017 |
| secondary_somatosensory | 0.043614 | 0.025 |
| judgment | 0.042499 | 0.012 |
| memory_retrieval | 0.040692 | 0.011 |
| repetition | 0.039148 | 0.043 |
| syntactic | 0.038496 | 0.036 |
| response_inhibition * | 0.035561 | <0.001 |
| planning * | 0.035438 | 0.002 |
| finger_movements | 0.033814 | 0.02 |
| control_processes * | 0.033256 | 0.001 |
| decision_making | 0.032696 | 0.018 |
| empathy | 0.031556 | 0.024 |
| naming | 0.030524 | 0.039 |
| visuospatial | 0.030258 | 0.014 |
| recall * | 0.029367 | 0.001 |
| reasoning * | 0.028662 | 0.003 |
| cognitive_processes * | 0.028345 | 0.001 |
| primary_somatosensory | 0.028121 | 0.038 |
| suppression | 0.027968 | 0.044 |
| recognition_memory * | 0.027899 | 0.001 |
| switching * | 0.027259 | 0.003 |
| sensation | 0.026812 | 0.022 |
| executive_control * | 0.026244 | 0.003 |
| stroop * | 0.026185 | 0.002 |
| tapping | 0.026088 | 0.026 |
| anticipation | 0.025814 | 0.027 |
| arithmetic * | 0.025289 | 0.004 |
| motor_imagery | 0.02437 | 0.041 |
| verbal_fluency | 0.024299 | 0.021 |
| memory_encoding | 0.0242 | 0.023 |
| memory_performance * | 0.024045 | 0.003 |
| recollection | 0.02267 | 0.03 |
| anxiety | 0.020941 | 0.019 |
| goal_directed | 0.020784 | 0.012 |
| salience | 0.020665 | 0.033 |
| incentive | 0.020656 | 0.047 |
| monitor | 0.020168 | 0.021 |
| encoding_retrieval | 0.020155 | 0.02 |
| emotional_responses * | 0.019672 | <.001 |
| stop_signal | 0.018812 | 0.024 |
| motivation | 0.018752 | 0.041 |
| mood | 0.018352 | 0.008 |
| motor_response | 0.018237 | 0.013 |
| nogo | 0.017641 | 0.006 |
| semantic_knowledge | 0.017611 | 0.008 |
| verbal_working * | 0.016959 | 0.004 |
| control_network | 0.016662 | 0.023 |
| attentional_control | 0.016465 | 0.038 |
| response_selection | 0.016357 | 0.01 |
| vulnerability | 0.016336 | 0.031 |
| performance_task * | 0.015489 | 0.001 |
| stress | 0.013305 | 0.014 |
| semantic_information * | 0.013227 | 0.001 |
| personality * | 0.013059 | 0.005 |
| sensory_information | 0.013055 | 0.048 |
| depression | 0.013034 | 0.014 |
| sustained_attention | 0.012684 | 0.012 |
| monetary_reward | 0.012594 | 0.042 |
| emotional_faces | 0.012512 | 0.038 |
| target_detection | 0.012458 | 0.014 |
| neurocognitive * | 0.012448 | <0.001 |
| pressure | 0.012348 | 0.018 |
| learning_task | 0.012313 | 0.023 |
| neutral_stimuli | 0.012013 | 0.006 |
| gain | 0.011947 | 0.018 |
| ongoing | 0.011787 | 0.033 |
| inhibitory_control | 0.011551 | 0.015 |
| awareness | 0.0113 | 0.048 |
| rhythm | 0.010911 | 0.008 |
| expectancy | 0.010526 | 0.015 |
| attention_deficit | 0.008371 | 0.01 |
| episode * | 0.00758 | 0.002 |
| emotional_valence | 0.007176 | 0.042 |
| emotional_information | 0.006504 | 0.016 |
| gambling | 0.006197 | 0.037 |
| major_depression * | 0.006169 | 0.002 |
| impulsivity | 0.00283 | 0.021 |

Note: Only functional terms that survived the permutation test (P < 0.05) are shown among 217 terms. * Terms that survived FDR correction (*q* < 0.05).

**Table S3. Functional annotation for the distinct edges**

| Functional terms of Neurosynth | Mean Co-activation Ratio | Permutation *P*-value |
| --- | --- | --- |
| selective attention * | 0.022728 | 0.024 |
| response selection | 0.019549 | 0.049 |
| learning task * | 0.01942 | 0.009 |
| ongoing | 0.015948 | 0.046 |
| attention deficit | 0.009105 | 0.049 |
| episode memory * | 0.008942 | 0.016 |
| hyperactivity | 0.00813 | 0.036 |
| impulsivity | 0.005327 | 0.008 |

Note: Only functional terms that survived the permutation test (P < 0.05) are shown among 217 terms. * Terms that survived FDR correction (*q* < 0.05).

**References for Supplementary Materials**

1 Charrad M, Ghazzali N, Boiteau V, Niknafs A. NbClust: an R package for determining the relevant number of clusters in a data set. J Stat Softw. 2014;61(6):1-36.
